# Numerical Optimization of Variable Blank Holder Force Trajectories in Stamping Process for Multi-Defect Reduction

**DOI:** 10.3390/ma17112578

**Published:** 2024-05-27

**Authors:** Feng Guo, Hoyoung Jeong, Donghwi Park, Geunho Kim, Booyong Sung, Naksoo Kim

**Affiliations:** 1 Department of Mechanical Engineering, Sogang University, Seoul 04107, Republic of Korea; guof@sogang.ac.kr (F.G.); ghy27@korea.ac.kr (H.J.); pdhwi93@u.sogang.ac.kr (D.P.); byseongs85@gmail.com (B.S.); 2 Department of Industrial and Management Engineering, Korea University, Seoul 02841, Republic of Korea; 3 R&D Center, ASAN Co., Ltd., Pureundeulpan-ro 826-4, Hwasung-si 18462, Republic of Korea; kgh7355@secoasan.com

**Keywords:** multi-objective optimization, defect predication, variable blank holder force trajectories, surrogate model methodologies

## Abstract

An intelligent optimization technology was proposed to mitigate prevalent multi-defects, particularly failure, wrinkling, and springback in sheet metal forming. This method combined deep neural networks (DNNs), genetic algorithms (GAs), and Monte Carlo simulation (MCS), collectively as DNN-GA-MCS. Our primary aim was to determine intricate process parameters while elucidating the intricate relationship between processing methodologies and material properties. To achieve this goal, variable blank holder force (VBHF) trajectories were implemented into five sub-stroke steps, facilitating adjustments to the blank holder force via numerical simulations with an oil pan model. The Forming Limit Diagram (FLD) predicted by machine learning algorithms based on the Generalized Incremental Stress State Dependent Damage (GISSMO) model provided a robust framework for evaluating sheet failure dynamics during the stamping process. Numerical results confirmed significant improvements in formed quality: compared with the average value of training sets, the improvements of 18.89%, 13.59%, and 14.26% are achieved in failure, wrinkling, and springback; in the purposed two-segmented mode VBHF case application, the average value of three defects is improved by 12.62%, and the total summation of VBHF is reduced by 14.07%. Statistical methodologies grounded in material flow analysis were applied, accompanied by the proposal of distinctive optimization strategies for the die structure aimed at enhancing material flow efficiency. In conclusion, our advanced methodology exhibits considerable potential to improve sheet metal forming processes, highlighting its significant effect on defect reduction.

## 1. Introduction

Stamping is a manufacturing process via high pressure applied to a sheet metal via a press and a die to form a desired shape [1,2,3]. This process is essential in industries like automotive and aeronautic component manufacturing, offering efficiency, low cost, and suitability for large-scale production [1,4]. However, some associated challenges including metal deformation, stress, and surface issues, can be addressed by adjusting the process and choosing the right materials [5,6].

**Figure 1:**
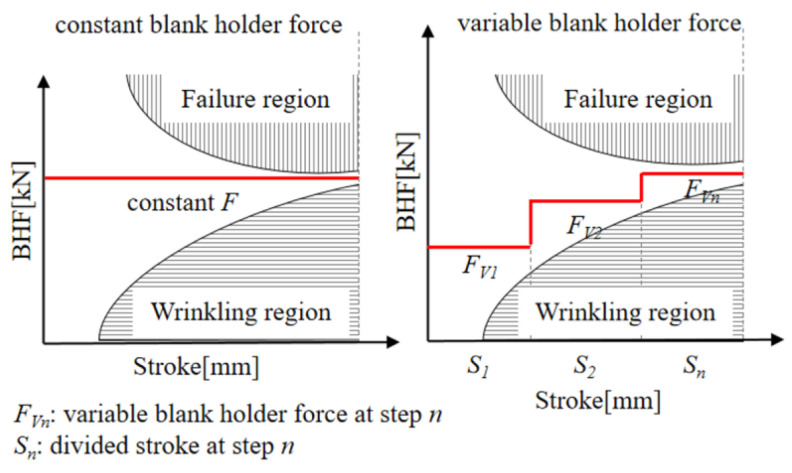
Schematic diagram of variable blank holder force trajectories indicating multi-defects.

Historically, a constant blank holder force (CBHF) was used in stamping processes, but this method usually results in material flow issues, causing wrinkling at low BHF and failures at high BHF, while increased tension and reduced bending moments might also worsen problems like springback [7,8]. The VBHF technique addresses these issues by allowing for the spatially and temporally variable forces on the blank holder, thus enhancing the control of the material flow [9,10]. The BHF trajectory is optimized to ensure smooth material flow, preventing local thinning and wrinkling [10,11]; a related basic schematic diagram is shown in Figure 1. Moreover, appropriate lubrication could increase sheet material flow to reduce defects [12]. Yet, excessively low friction might result in unintended issues like tearing or excessive thinning [13,14]. Moreover, optimizing stamping quality requires precise adjustment of the friction coefficient, which could be influenced by lubricant application position. However, determining the lubrication application position is obtained through continuous trial and error.

Research in recent years has been leaning toward advanced optimization methods, as listed in Table 1. Neural network cooperative interaction with genetic algorithms was used by Srirat et al. [15], Li et al. [16], Kitayama et al. [17,18,19], and Tran et al. [20]. However, some more practical and in-depth conclusions need to be drawn, instead of just a result parameter, to help engineering adjust process parameters in specific cases. Researchers, including Feng et al. [21,22], Zhai et al. [23], Xie et al. [24], Taşkın et al. [25], Jiang et al. [26], Yu et al. [27], and Guo et al. [10], have enriched this field by merging an area of design techniques incorporating the latest developments. Implementing these advanced computational techniques in the context of sheet metal forming represents a convergence of machine learning, optimization algorithms, and computational physics, but promising substantial addresses of the complex, nonlinear, and stochastic nature of the metal forming process need more substantial results and discussion. Moreover, advanced yield criteria should be used to accurately evaluate the anisotropy plane-stress state to ensure the accuracy of the analytical and numerical calculations.

Nevertheless, extensive expertise in defining the scope of learning material selection, data selection, and preprocessing is required, but it consumes a lot of time and resources [28,29]. Using models with low learning ability is not suitable for complex data processing and accurate predictions [30]. Additionally, excessive use of training materials that force the model to memorize training data rather than learning basic patterns can reduce the model’s ability to predict new data [31]. In addition, predictions can be obtained from a learning model, but it is not easy to derive a generalized, interpreted mathematical model for the data. Consequently, it is essential to develop an interpretable process modeling and optimization method that incorporates physical principles into the optimization method and changes the optimization process from a black box to a glass box [10,32].

Despite significant progress, as highlighted by Xie et al. [24], remain about quality fluctuations caused by material and process parameter variations. It is essential to recognize that even slight deviations in the forming process could lead to significant alterations in results. Therefore, the innovative integration of Response Surface Methodologies (RSMs) with Monte Carlo simulation (MCS) by Gantar et al. [33] laid the groundwork for predicting system responses under process parameters including variable BHF conditions. Expanding upon this groundwork, investigations by Zhang et al. [34,35] and Marretta et al. [36,37] elucidated multi-objective explanations and effectively dealt with the complexities associated with process variabilities.

**Figure 2:**
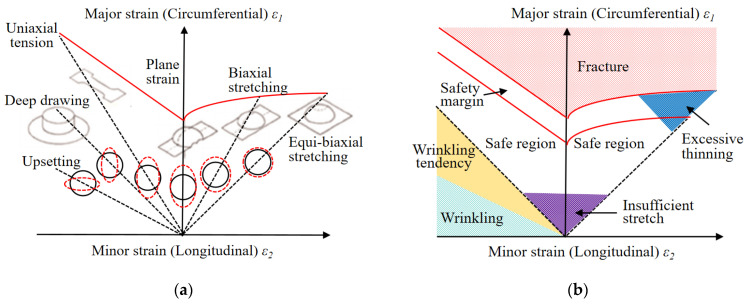
The framework of forming quality and intelligent optimization performance. (**a**) schematic representation, (**b**) various defects and delineated safe forming zone of the FLD.

Additionally, the estimation of material processability is crucial in assessing the workability of sheet metals and identifying product issues in forming processes. The Forming Limit Diagram (FLD) was commonly utilized in experimental settings to assess the formability of manufactured components [10,15,17,18,19,23,24]. Keeler and Backofen [38] and Goodwin [39] are acknowledged as trailblazers in the initial development of the FLD. Figure 2a,b present a schematic representation of the FLD and illustrate various defects and delineate a safe forming zone, respectively. Typically, FLD graphically represents the major in-plane strain and the minor in-plane strain on the vertical or horizontal axis. Moreover, the Forming Limit Curve (FLC) is a delimitation on the FLD that divides between safe and unsafe levels of strains. However, accurately determining this curve requires understanding the material’s behavior under various conditions, in which the variability in these factors makes the FLC calculation a sophisticated process requiring precise experimental data and advanced mathematical modeling. Moreover, creating FLDs for specific materials and thicknesses requires expensive experiments, causing challenges for most producers and those using diverse materials.

Addressing the mentioned challenges often requires a multi-disciplinary approach, combining material science, mechanical engineering, and computational modeling, to enhance the predictive ability of FLDs. The experimental procedure for measuring strain from gridded samples is costly and laborious and requires both skill and care to accurately judge the FLC. Therefore, analytical and numerical methods for determining the FLC have been developed as listed in Table 2. Researchers considered Punch stroke, oil pressure [30], forming rates [40], and chemical composition with temperature conditions [28] to train artificial intelligence models, while some other authors mainly considered material properties such as YS, UTS, EU, EL, etc., and supplemented them with simple engineering conditions such as *R*, *n*, *t*, etc., to predict FLC [29,41,42]. The current work utilized appropriate experimental data and advanced machine learning modeling to enhance predictability, enabling more efficient and cost-effective manufacturing practices by reducing the reliance on extensive physical testing. Thus, utilizing the GISSMO model to understand the response of materials under different conditions of stress and deformation is critical for predicting failure points accurately.

Although the potential of VBHF in the manufacturing process was recognized as listed in Table 1, there remains a comprehensive investigation into its inherent variability and integration. By utilizing more advanced characterization techniques, meticulous analytical methods, and more intelligently computational approaches, our objective was to elucidate the intricate relationship between processing techniques and material properties. These intricate relationships were constructed by the previous studies but deficiently in terms of visualization. A key aspect of our study involved the identification of defects at both the numerical processing and structural levels, as these defects play a critical role in influencing mechanical properties. These mechanical properties, in turn, impact the innovative processing control that follows, ultimately enhancing the overall performance of materials and structures. Our study aimed to make original and significant advancements in various key areas:

- The FLD predicted by a machine learning algorithm based on the GISSMO damage model provided an advanced rigorous evaluative framework and applied an overall assessment of sheet failure in the forming process.
- Deep neural network (DNN) modeling could model complex nonlinear relationships between process parameters and the resulting product quality, facilitating the rapid evaluation of different parameter sets.
- The nondominated sorting genetic algorithm-II (NSGA-II) could adjust process parameters to minimize multi-defects simultaneously.
- Monte Carlo simulation (MCS) techniques were used to model the probability of different outcomes that have uncertainty, facilitating the assessment of the robustness of selected process parameters.
- Subsequently, statistical methodologies grounded in material flow analysis were applied, accompanied by the proposal of distinctive optimization strategies aimed at enhancing material flow efficiency.

This research introduced a framework for multi-objective optimization that focuses on mitigating defects related to failure, wrinkling, and springback. The core of this inquiry was the utilization of surrogate model methodologies based on the DNN-GA-MCS amalgamation of delivering unparalleled accuracy in addressing complex parameter interactions. The DNN incorporated an index from simulation outcomes and defect evaluations simultaneously, while through the GA, it obtained Pareto-optimal solutions iteratively to minimize multiple defects. After that, the MCS evaluated solution uncertainty to enhance process reliability. Simulation models were utilized to evaluate formability and quantify the defect severity of failure, wrinkling, and springback. Following a rigorous optimization process, the intricate parameters governing VBHF were meticulously censored, leading to reductions in defects and indicating enhanced formability. Subsequently, statistical methodologies grounded in material flow analysis were applied, accompanied by the proposal of distinctive optimization strategies aimed at enhancing material flow efficiency.

## 2. Finite Element Analysis Model and Design Variable Definition

### 2.1. Oil Pan Finite Element Model

**Figure 3:**
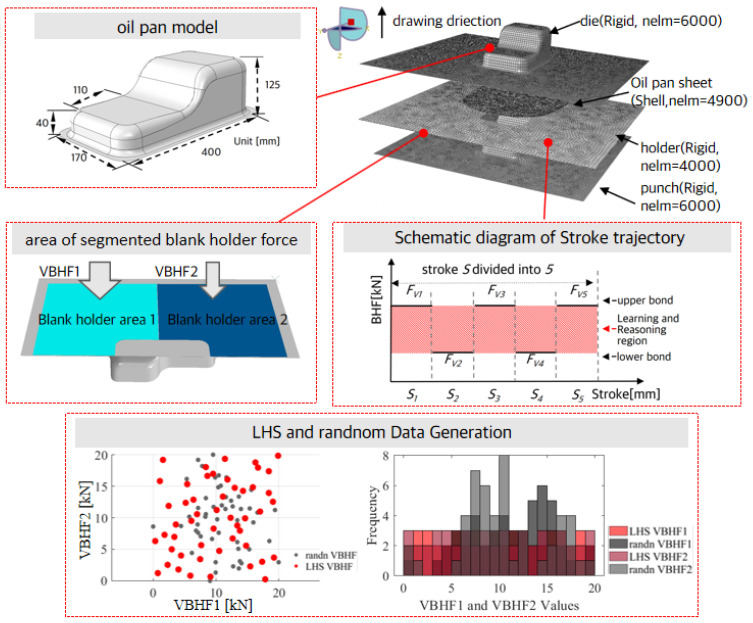
Schematic diagram of assembly FEA model with oil pan stamping part, segmented VBHF region, design variables of the VBHF, and LHD sampling scheme.

This study investigates a stamping process applied to an oil pan model within a finite element analysis (FEA). The FEA model assembly schematic diagram, the dimensions of the oil pan stamped product part, and the region of the two BHF variables are depicted in Figure 3. Rigid elements were employed to simulate the states of the punch, blank holder, and die. The blank elements incorporated Belytschko and Tsay shell elements with seven integration points distributed through a 1 mm thickness. To ensure the precision of subsequent optimization designs, a grid independence test was conducted. Surface contact conditions between interfaces were governed by a friction coefficient, serving as a penalty coefficient of 0.08 considering a similar situation to the literature cited in [9,17,18]. The yield function evaluated in the ABAQUS VUMAT user subroutine was defined by employing the Yld2004-18p coefficients of the proposed aluminum material outlined in Table 2.

The VBHF was applied while the punch descended at a rate of 300 mm/s. The total stroke was separated by five sub-stroke steps, each maintaining a constant BHF along its trajectory. Then, the VBHF of each sub-stroke step was used as design variables. The delimited lower and upper bounds of the design variables were defined as 10 kN and 25 kN, and the maximum total magnitude of the two regions was defined as 30 kN for the design constant. The optimal sampling points were created by using the Latin hypercube design (LHD) referenced from [18,19], upon which a deep neural network (DNN) model was established to study the relationship between variable blank holder force (VBHF) and multi-defects in the stamping process. Considering the 10-dimensional design space is difficult to visualize, we illustrated the LHD sampling scheme shown in Figure 3 by extracting 30 sample points in a two-dimensional design space.

### 2.2. Material Properties

**Figure 4:**
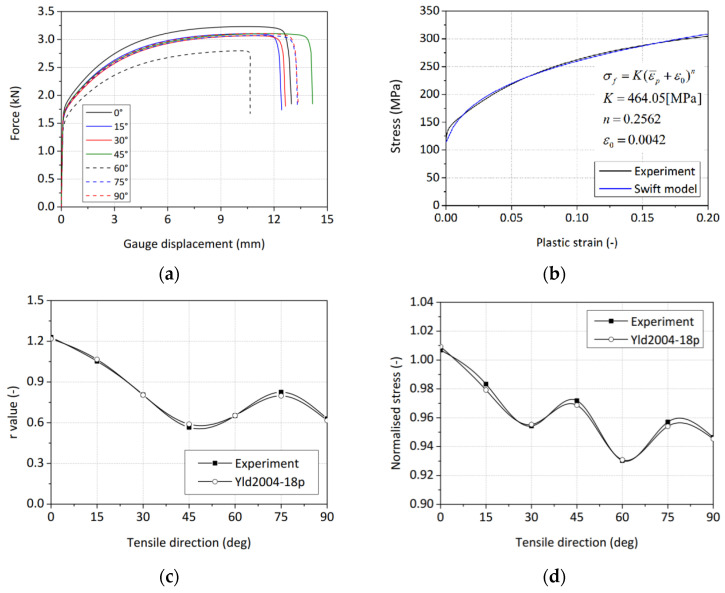
Anisotropic material characteristics of AA6014-T4 alloy of (**a**) force-displacement curves, (**b**) true stress–strain curves, (**c**) R-value data, and (**d**) anisotropy calibration results.

The studied material focused on a 1.0 mm thickness aluminum alloy AA6014-T4 sheet; a description is mentioned in [10]. The test setup for uniaxial tensile experiments included a horizontal-type tensile testing machine and a video extensometer. Strain fields were calculated using the digital image correlation (DIC) method through X-Sight (ALPHA 2022 SP1) software, analyzing image sequences. The UT specimen configurations followed ASTM E8 standards, with loading applied in 15° increments from 0~90° of the rolling direction. Loading test was performed at a constant rate of 1 mm/min until instability. Figure 4 illustrates the anisotropic material properties based on the Yld2004-18p yield function.

To understand and predict material behavior in diverse forming process parameters, advanced characterization used the Yld2004-18p yield function introduced by Barlat et al., 2005 [43], which accurately captures the anisotropic behavior of the alloy through 18 parameters, allowing accurate predictions of mechanical responses under various loading conditions, thereby enhancing structural analysis accuracy. This comprises a mathematical expression, as shown below:

(1) $$\begin{array}{c} \emptyset =4{\bar{\sigma }}^{a}={\left|{\tilde{S}}_{1}^{\prime }-{\tilde{S}}_{1}^{\prime \prime }\right|}^{a}+{\left|{\tilde{S}}_{1}^{\prime }-{\tilde{S}}_{2}^{\prime \prime }\right|}^{a}+{\left|{\tilde{S}}_{1}^{\prime }-{\tilde{S}}_{3}^{\prime \prime }\right|}^{a}+{\left|{\tilde{S}}_{2}^{\prime }-{\tilde{S}}_{1}^{\prime \prime }\right|}^{a}\\ +{\left|{\tilde{S}}_{2}^{\prime }-{\tilde{S}}_{2}^{\prime \prime }\right|}^{a}+{\left|{\tilde{S}}_{2}^{\prime }-{\tilde{S}}_{3}^{\prime \prime }\right|}^{a}+{\left|{\tilde{S}}_{3}^{\prime }-{\tilde{S}}_{1}^{\prime \prime }\right|}^{a}\\ +{\left|{\tilde{S}}_{3}^{\prime }-{\tilde{S}}_{2}^{\prime \prime }\right|}^{a}+{\left|{\tilde{S}}_{3}^{\prime }-{\tilde{S}}_{3}^{\prime \prime }\right|}^{a} \end{array}$$

where the index “*a*” in the yield function was determined as 8 based on the material’s crystallographic structure, and the computation of these parameters involved an optimization process using mechanical properties detailed in Figure 4 and Table 3.

## 3. Design Optimization

### 3.1. Design Optimization Problem Description

This section addresses the mitigation of forming defects in metal stamping through a multi-objective optimization approach. The standard formulation for multi-objective design optimization is articulated by Miettinen [44]. Under this framework, *obj* depicts the objective function encompassing failure, wrinkling, and springback. The design variables are represented as *x_i_* and have lower and upper boundaries, *x_i_^L^* and *x_i_^U^*. The total design variable number is *n*, and *f*(*x*) is the objective function to be minimized, while constraints are denoted as *g_j_*(*x*), along with the constraint count *n_con_*.


(2) $$\begin{array}{c} \mathrm{Minimize}\;{\mathrm{obj}}_{f},\;{\mathrm{obj}}_{w},\;{\mathrm{obj}}_{s},\\ \mathrm{Subject}\;\mathrm{to}\;{X_{i}}^{L}\;\leq \;X_{i}\;\leq \;{X_{i}}^{U}\;\;i\;=\;1,2,\;\ldots ,\;n\\ g_{j}(x)\;\leq \;0\;\;j\;=\;1,2,\;\ldots ,\;n_{\mathrm{con}} \end{array}$$

### 3.2. Objective Functions

**Figure 5:**
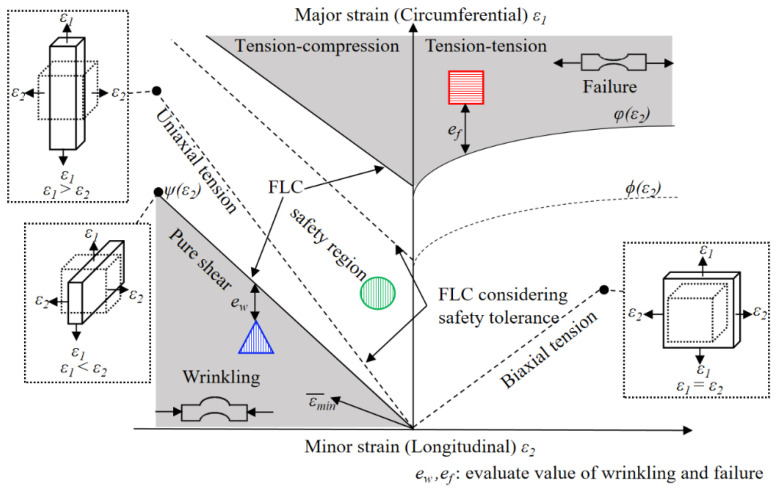
Schematic diagram of objective functions based on FLD.

Within this study, multi-objective optimizations were conducted by assessing various objective functions, with a specific focus on failure, wrinkling, and springback. Initially, strain values were gathered from elements of the simulated sheet component. Subsequently, the locally correlated variable related to the defect response was assessed by the Forming Limit Diagram (FLD) and utilized as objective functions in the context of the DNN-GA-MCS strategy. Criteria derived from the FLD were used to assess formability and identify forming defects in sheet product parts. The objective functions were then determined using these criteria, with the goal of minimizing the occurrence of defects during the optimization process, as depicted in Figure 5.

Instances where the major strains of components overstep the FLD line ϕ(ε_2_) or outdistance the safety boundary line ϕ(ε_2_) may lead to failure in the relevant areas of the component. Failure assessment criteria were established by evaluating the distance between the major strain of given elements on the FLD. Consequently, the objective function for failure was defined as the sum of squares of deviations for all points and is presented as follows:

(3) $${Obj}_{f}=\left\{\begin{array}{cc} \sum_{i=1}^{nelm}{\left(e_{f}^{i}\right)}^{2}=\sum_{i=1}^{nelm}{\left(\varepsilon_{1}^{i}-\varphi (\varepsilon_{2}^{i})\right)}^{2} & \varepsilon_{1}^{i}>\varphi (\varepsilon_{2}^{i})\\ 0 & \varepsilon_{1}^{i}\leq \varphi (\varepsilon_{2}^{i}) \end{array}\right.$$

Similarly, wrinkling assessments were developed by considering the distance between the major strain of a specified point and the Wrinkling Limit Curve (WLC) represented by the line ψ(ε_2_). The formulation of the wrinkling objective function is outlined in the subsequent equation:

(4) $${Obj}_{w}=\left\{\begin{array}{cc} \sum_{i=1}^{nelm}{\left(e_{w}^{i}\right)}^{2}=\sum_{i=1}^{nelm}{\left(\psi (\varepsilon_{2}^{i})-\varepsilon_{1}^{i}\right)}^{2} & \varepsilon_{1}^{i}<\psi (\varepsilon_{2}^{i})\\ 0 & \varepsilon_{1}^{i}\geq \psi (\varepsilon_{2}^{i}) \end{array}\right.$$

Low plastic stretching can cause slight elastic deformation leading to springback. Therefore, the springback criterion is described by the distance between the major strain of each point and the corresponding minimized effective plastic strain, as follows:

(5) $${Obj}_{s}=\left\{\begin{array}{cc} \sum_{i=1}^{nelm}{\left(e_{s}^{i}\right)}^{2}=\sum_{i=1}^{nelm}{\left(({\bar{\varepsilon }}_{min}^{i})-({\bar{\varepsilon }}^{i})\right)}^{2} & {\bar{\varepsilon }}^{i}>{\bar{\varepsilon }}_{min}^{i}\\ 0 & {\bar{\varepsilon }}^{i}\leq {\bar{\varepsilon }}_{min}^{i} \end{array}\right.$$

## 4. Machine Learning Algorithm for Prediction of Forming Limit Diagram (FLD)

This study introduced a machine learning algorithm designed to predict the FLD for stamping processes. The training datasets for this algorithm were derived from fracture locus calculations conducted using the GISSMO damage model. This model was well regarded for its capability to accurately predict material failure under various loading scenarios, providing extensive data on stress states and corresponding strains at the point of fracture. By integrating these comprehensive datasets into a machine learning framework, the algorithm was trained to identify patterns and predict formability limits under various forming conditions. This methodology not only enhanced the precision of FLD predictions but also optimized the FLD prediction process.

### 4.1. GISSMO Damage Model

The Generalized Incremental Stress-State-Dependent Damage Model (GISSMO) is a theoretical framework designed to forecast the instability, softening, and fracture behaviors of metallic materials [45,46,47]. Initially introduced and enhanced by [48,49,50,51], the GISSMO model primarily employs a nonlinear method for damage accumulation to establish its damage criterion. Anderson et al. [52] utilized hybrid experimental-numerical techniques, employing butterfly specimen tests, to verify the GISSMO model’s accuracy in predicting damage evolution and structural failure.

In the GISSMO fracture criterion, crack generation is allowed for any path, and the crack generation is determined by the damage factor D, the damage evolution is defined as follows:

(6) $$\dot{D}={n\left(\frac{\varepsilon^{p}}{\varepsilon_{f}\left(\eta \right)}\right)}^{n-1}\frac{{\dot{\varepsilon }}^{p}}{\varepsilon_{f}\left(\eta \right)}$$

where the dot above a variable indicates its time derivative. D is the damage value, ε_p_ is the effective plastic strain, n is the model coefficients, η is the stress triaxial ratio, and ε_f_(η) is the fracture strain as a function of the stress triaxial ratio. When D reaches 1.0, the element cannot sustain stress and is removed.

Additionally, the GISSMO model enables the coupling of stress and damage within a single element. This coupling is facilitated through the introduction of an instability parameter F. The F evolution, a variable that couples the stress and damage values, is defined as follows:

(7) $$\dot{F}={\left(\frac{n}{\varepsilon_{crit}\left(\eta \right)}\right)F}^{\left(1-1/n\right)}{\dot{\varepsilon }}^{p}$$

where ε_crit_(η) is the critical strain as a function of the stress triaxial ratio. When F attains 1, the stress of the element decreases as

(8) $$\dot{\sigma }=\sigma [1-{\left(\frac{D-D_{crit}}{1-D_{crit}}\right)}^{m}]$$

where σ represents the stress modified by damage. D_crit_ is the critical damage threshold at which F reaches 1, and m is the falling factor indicating the material softening rate.

### 4.2. Fracture Experiment

Within this study, AA6014-T4 alloy sheets with 1 mm thickness were utilized. To achieve varied stress triaxialities, test specimens consisting of smooth, R5 notch, R15 notch, 0° shear, 45° shear, and Nakajima test specimens with widths of 200 mm were employed. Based on the Bridgman formula, the stress triaxiality value was determined to range from 0 to 0.5 [53,54,55,56]. The designs of these specimens were adapted from those detailed by Andrade et al. [51], with three replicates conducted for each test condition. Strain measurements during the tests were performed using digital image correlation (DIC). The tensile testing speeds were set at 0.1 mm/min for shear samples and 2 mm/min for the other types of specimens.

**Figure 6:**
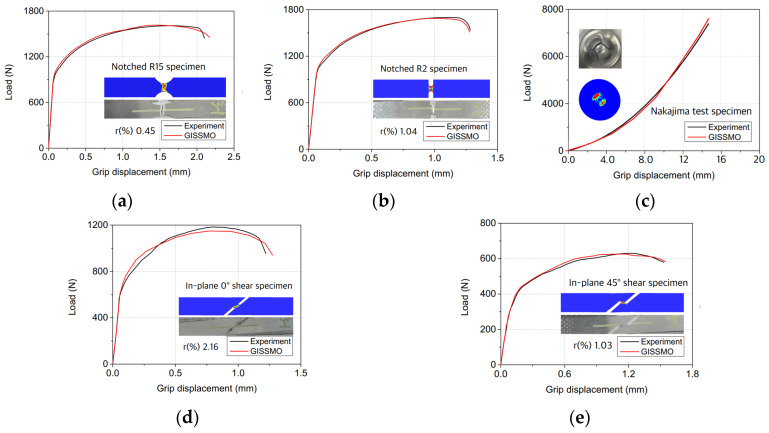
Predicted and measured force-displacement data for assessing the Forming Limit of Sheet Materials, displayed sequentially from left to right are the specimens for (**a**) Notched R15, (**b**) Notched R2, (**c**) Nakajima test, (**d**) in-plane 0° shear, and (**e**) in-plane 45° shear.

A combined experimental and numerical methodology was employed to determine the fracture strain by identifying the fracture point on the experimental load-displacement curve. The equivalent plastic strain, derived from numerical analyses correlating with the observed displacement, was used to quantify the fracture strain. Based on the fracture locus and the optimized values of n and m, a GISSMO model for the AA6014-T4 material was developed. This study yielded the optimized parameters of n = 5.4 and m = 8.2. Figure 6 displays both the experiment-measured and FEA-predicted load-displacement curves, illustrating that the established damage model estimates ductile fracture under various stress triaxialities with a high accuracy, after calibrating the grid correlation. Both observed and predicted fracture mode and fracture strain demonstrated strong concordance.

### 4.3. Fracture Locus

Fracture tests were conducted on specimens designed to reflect different stress states in aluminum alloy sheets. The findings were integrated with finite element analysis simulations to fine-tune the coefficients of the GISSMO damage parameters. The fracture strain was determined for six specific stress states: standard smooth uniaxial tension, notched tension (R2/R9), shear at 0°/45° angles, and Nakajima 200 mm specimens.

In 1990, Mohr and Coulomb [57] introduced the Mohr–Coulomb (MC) crack growth model, which was further elaborated by Mohr in a subsequent study to formulate the Hosford–Coulomb (HC) model [58]. This model was designed to forecast fracture development under non-proportional loading conditions [54].

(9) $$\begin{array}{c} \varepsilon_{f}\left(\eta \right)={b\left(1+c\right)}^{1/n}\{{\left[\frac{1}{2}{\left(f_{1}(\bar{\theta })-f_{2}(\bar{\theta })\right)}^{a}+{\left(f_{2}(\bar{\theta })-f_{3}(\bar{\theta })\right)}^{a}+{\left(f_{1}(\bar{\theta })-f_{3}(\bar{\theta })\right)}^{a}\right]}^{1/a}\\ {+c[2\eta +f_{1}(\bar{\theta })+f_{3}(\bar{\theta })]\}}^{-1/n} \end{array}$$

where η is denoted by stress triaxiality, a, b, and c represent material parameters, the work hardening exponent is indicated by n, and θ is the trigonometric function related to the lord angle parameter as

(10) $$\begin{array}{c} f_{1}(\bar{\theta })=\frac{2}{3}cos[\frac{\pi }{6}(1-\bar{\theta })];\\ f_{2}(\bar{\theta })=\frac{2}{3}cos[\frac{\pi }{6}(3+\bar{\theta })];\\ f_{3}(\bar{\theta })=-\frac{2}{3}cos[\frac{\pi }{6}(1+\bar{\theta })]; \end{array}$$

For the plane strain, the Lode angle parameter can be determined as

(11) $$\bar{\theta }=1-\frac{\pi }{6}=1-\frac{2}{\pi }arccos[-\frac{27}{2}\eta (\eta^{2}-\frac{1}{3})]$$

**Figure 7:**
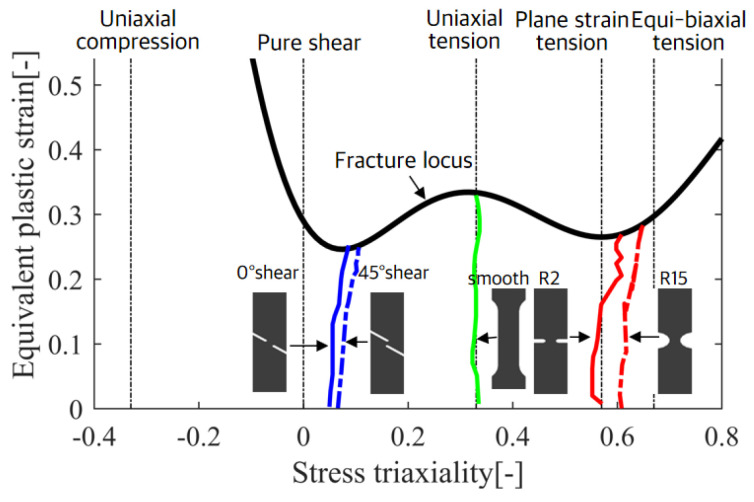
Fracture characteristic locus of stress triaxiality distribution curves.

Utilizing spline interpolation, a characteristic curve of fracture strain was established as shown in Figure 7. Observations of the variation in the stress triaxiality ratio at the failure section highlighted the damage effect, indicating that the material exhibits different mechanical behaviors under diverse stress scenarios. As the equivalent plastic strain increases, observe how the stress triaxial ratio evolves for each stress state. The calculated average stress triaxial ratios for these states are 0.08, 0.15, 0.33, 0.41, 0.57, and 0.64.

## 5. Surrogate Model Methodologies Based on DNN-GA-MCS Strategy

**Figure 8:**
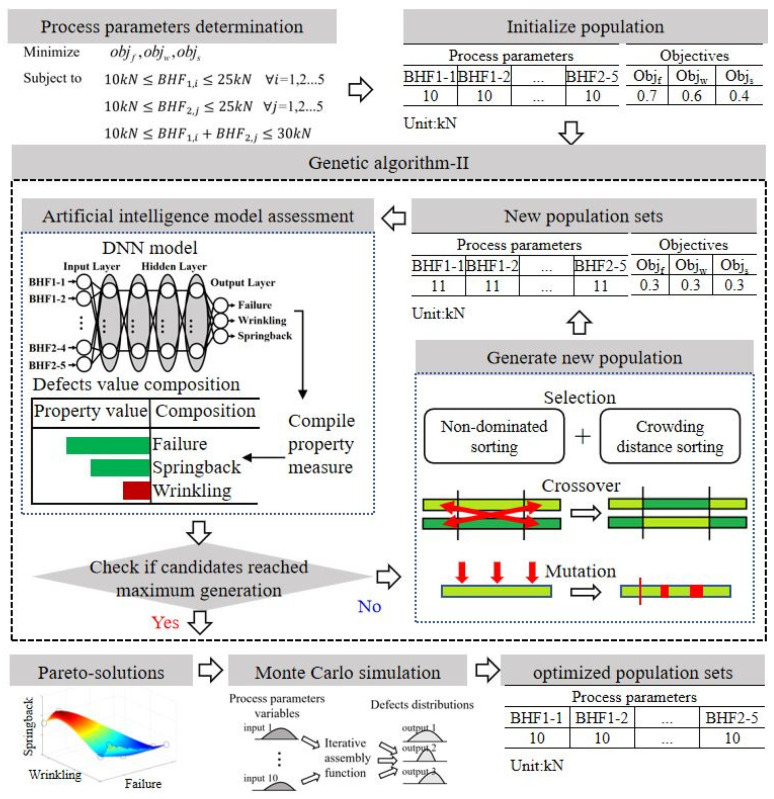
Schematic diagram of VBHF multi-objective optimization problem.

In this study, the proposed surrogate model methodologies based on the DNN-GA-MCS strategy for the multi-objective optimization of the stamping process integrate machine learning and optimization methodologies to reduce the likelihood of failure, wrinkling, and springback defects. The comprehensive depiction of the DNN-GA-MCS process is encapsulated in Figure 8. Surrogate models provided efficient approximations for complex systems, enabling faster optimization and global sensitivity analysis while reducing computational costs. The DNN was trained to approximate defect probabilities contingent upon various VBHF trajectories. Subsequently, the objectives after approximation were utilized as inputs for the NSGA-II to gain Pareto-optimal sets, optimizing the processing design. The resultant optimal trajectories of the VBHF were subsequently analyzed through MCS to evaluate their frequency properties under uncertainty. The model constructions are detailed in Appendix B, Appendix C, and Appendix D, respectively.

Ultimately, the optimized VBHF trajectories were further validated through the Finite Element Method to understand and predict material behavior in diverse forming process parameters. The core technology of this research is the defects operating at the numerical processing structural level, which could play an essential role in determining mechanical properties. Numerical results highlighted the practical significance obtained by the DNN-GA-MCS approach in complex engineering region applications, especially the practical significance of providing robust and reliable process parameters.

## 6. Numerical Results

This section presented and discussed the results obtained by applying the proposed method in the stamping processes. Firstly, the DNN model was validated, and an approximate surface was generated to explain the relationship between defects and VBHF trajectories. Subsequently, the NSGA-II was used for the analysis of Pareto-optimal sets, and the efficiency of this method in reducing defects was demonstrated. The MCS evaluated the uncertainty of these solutions, emphasizing the enhancement of process reliability. Subsequently, the optimized VBHF trajectories were verified using FEA and evaluated by the FLD based on the GISSMO damage model.

### 6.1. Forming Limit Diagram Prediction

**Figure 9:**
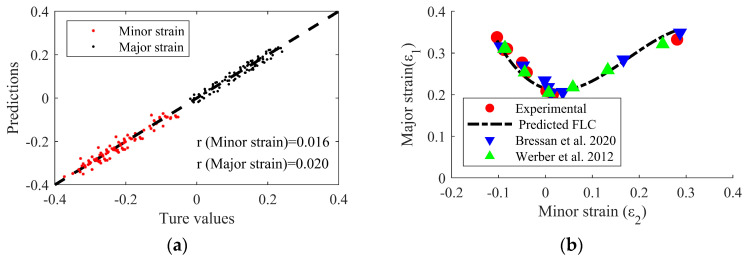
(**a**) Accuracy of minor strain and major strain distribution and (**b**) predicted FLDs for AA6014-T4 for using machine learning model and experiment data collected from literature [59,60] to validate proposed model.

Figure 9a shows the accuracy of major and minor strain distribution, and Figure 9b shows the predicted FLDs for AA6014-T4 for validating the proposed model using the machine learning model and the experimental data collected from the literature. The results of comparing the major and minor strain using the damage model coefficients derived from the experiment data and derived by machine learning are shown in Figure 9b. Employing these findings, more precise predictions could be facilitated by generating a forming limit curve using artificial intelligence techniques. Major and minor strain learning accuracy results in the high values of 0.016 and 0.020. Building the derived major and minor strains into a molding limit curve yielded a curve with high accuracy compared to published literature results [59,60]. The RMSE value was higher by 0.009 compared to the curve derived using the machine learning model and the literature results. It was more accurate when used to assess plate fracture and instability and localized damage.

### 6.2. Approximation of VBHF Result via Deep Neural Network (DNN) Modeling

**Figure 10:**
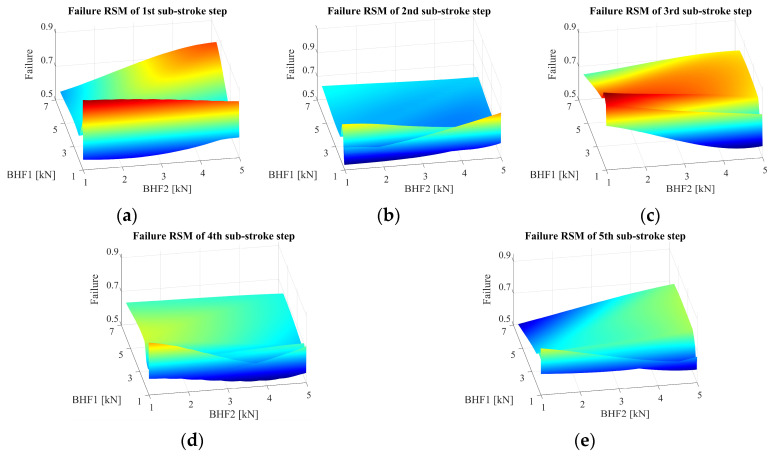
Approximate RSM model for the VBHF1 and VBHF2 trajectories with failure defects, depicting the defect relationship across sub-stroke steps 1 to 5. Figure (**a**–**e**) illustrate unique trajectories for each sub-stroke. Each sub-figure represents a distinct trajectory, illustrating the variations in the models under consideration.

Through ABAQUS, simulations based on sampling points were generated by the Latin hypercube design (LHD), and 150 groups of VBHF trajectories were obtained for experimental runs. Utilizing these design groups, training datasets were constructed, revealing clear nonlinear interactions among the defined process variables with a defect. Considering the huge amount of data for 10-dimensional design space visualization, we illustrated the graph multi-defect RSM of each sub-stroke step in trajectory in three-dimensional design space. Figure 10 illustrates that as the approximate model for the VBHF trajectories with three defects of the defect relationship of each sub-stroke step. This showed that combined with LHD design, the inherent mechanism of machine learning algorithms could switch complex process parameters containing multiple trajectories into interpretable mathematical models. The industrial problem of finding optimal trajectories therefore became understanding the mathematical model constructed in this study by exploring the optimal solution of the split VBHF coupling with the response surface. That is, finding the lowest multi-defect optimization by adjusting process parameters within the range of visualization.

**Figure 11:**
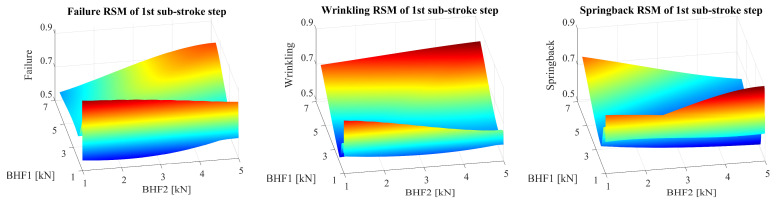
Approximate RSM model for the VBHF trajectories with three defects, depicting the defect relationship across sub-stroke steps 1.

In the framework of the Response Surface Methodology (RSM) applied to the VBHF trajectories, three defects were examined as shown in Figure 10. Each individual defect is analyzed by every sub-stroke step, exhibiting the unique trajectories associated with each defect. The figures are divided into sub-figures and each sub-figure represents a distinct defect. This arrangement effectively illustrates the variations present in the models under investigation. Notably, the sub-stroke step 1 has a greater influence on the three-species coupling than the sub-stroke step 2. The failure RSM related to sub-stroke steps 1 and 3 and failure related to sub-stroke steps 2, 4, and 5 showed opposite characteristics in Figure 11.

### 6.3. VBHF Optimization Result via Nondominated Sorting Genetic Algorithm-II (NSGA-II)

**Figure 12:**
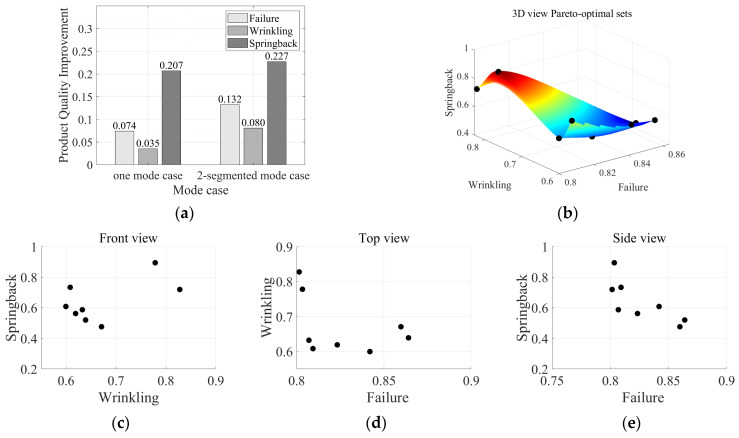
(**a**) Defect optimization result comparison with training set average values of one-mode case and two-segmented mode case Pareto-optimal set surface with (**b**) 3D view space, (**c**) front view, (**d**) top view, and (**e**) side view.

NSGA-II was utilized to identify the optimal VBHF trajectories with the objective of reducing all three defects simultaneously. Figure 12 shows the results obtained by NSGA-II, where each point represents the Pareto optimal sets. These findings proved that the suggested methodologies based on the DNN-GA-MCS strategy approach effectively produced optimal variables considering global and local optimization while minimizing the three defect possibilities.

When the defect optimization results are compared with the training set average values, all defects including failure, wrinkling, and springback are reduced within the Pareto-optimal set range. As shown in Figure 12a, in the case of two-segmented mode VBHF case application, the total aggregate quantity of VBHF is reduced by 14.07%, and when compared with the average value of training sets, the improvements of 18.89%, 13.59%, and 14.26% are achieved in failure, wrinkling, and springback, respectively. Meanwhile, compared to the average value of the objective function of the non-segmented and two-segmented training sets, the two-segmented case showed better performance in terms of total value by 12.62%.

### 6.4. Pareto Chart Results via Monte Carlo Simulation (MCS)

**Figure 13:**
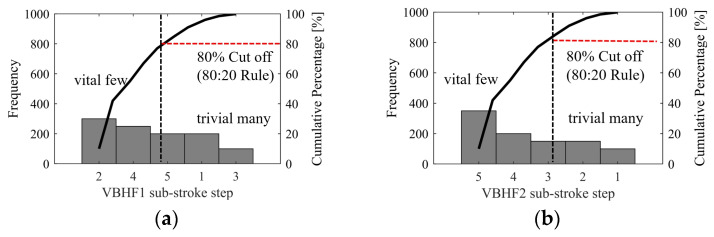
Pareto chart of types of VBHF trajectory sub-stroke step frequencies of trajectories of (**a**) VBHF1 and (**b**) VBHF2, vital few sub-stroke steps that warrant most attention are disparate as sub-stroke steps 2 and 4 and sub-stroke steps 5 and 4, respectively.

Figure 13a,b illustrate a Pareto chart detailing the frequencies of sub-stroke steps for various VBHF trajectories. A comprehensive review of VBHF trajectories and their sub-stroke step frequencies was conducted to ascertain the cumulative total of defects and their corresponding frequencies. The data were initially gathered on a tally sheet, after which they were organized in descending order of frequency and displayed on a Pareto chart template. This chart features both bars and a line: the bars represent individual values in descending order, while the cumulative total for the sample is depicted by a curved line. An 80% cutoff line is included to demonstrate the application of the 80/20 rule, indicating the critical few factors that require the most attention, situated beneath this line. From this, the sub-stroke steps for the trajectories of VBHF1 and VBHF2 with the most attention are disparate as sub-stroke steps 2 and 4 and sub-stroke steps 5 and 4, respectively. In contrast, the sub-stroke steps where large manufacturing tolerances can be used are sub-stroke steps 1 and 3 and sub-stroke steps 1 and 2 of VBHF1 and VBHF2, respectively. This outcome offers valuable guidance for engineers regarding the precise establishment of tolerance grades.

### 6.5. Numerical Optimization Results

**Figure 14:**
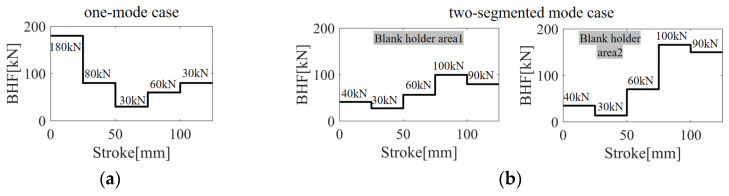
The optimal VBHF trajectories for different configurations: (**a**) the one-mode case and (**b**) the two-segmented mode case.

Figure 14 depicts the optimal VBHF trajectories for different configurations: (a) the one-mode case and (b) the two-segmented mode case. In the one-mode case, the blank holder area was treated as a unified whole, whereas in the two-segmented mode case, it was divided into two distinct sections: Blank Holder Area 1 and Blank Holder Area 2. It is important to note that with a constraint of the total VBHF amount below 300 KN, the two-segmented mode case is reduced by 60.51 kN, which is 14.07% of the total reduction in the total BHF of one mode case.

**Figure 15:**
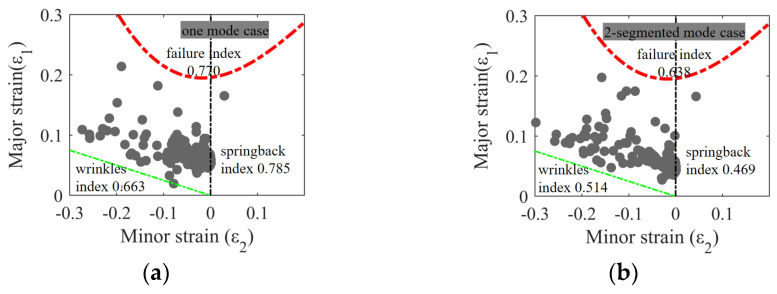
The FLD result comparison of the optimal-case simulation result of (**a**) the one-mode case and (**b**) the two-segmented mode case.

The optimal-case FLD is shown in Figure 15, in which all major strain values are within the safe region. Compared with the one-mode case, the wrinkling objective function value decreased by 12.47%. Furthermore, the values of the springback and failure objective functions were reduced by 10.25% and 7.14%, respectively, indicating substantial improvements in solving multi-defect reduction variation issues. Furthermore, compared with the similar structure stamping results shown in the literature [9,17,19], the FLD distributions are more uniform in the low plastic stretching regions due to the optimization of springback. In addition, from the above results, this optimization method is a feasible solution to the conflict relationship between multi-defects.

**Figure 16:**
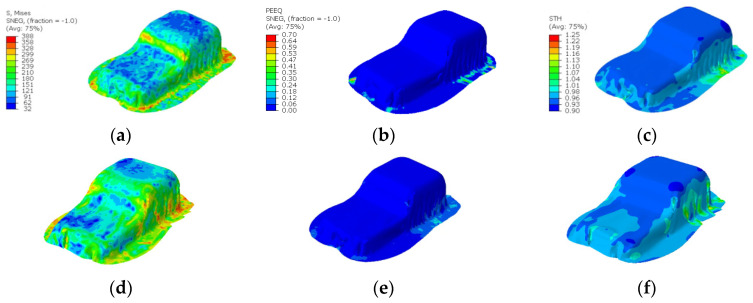
Numerical optimization results of stress distribution, strain distribution, and thickness distribution of (**a**–**c**) one-mode BHF case and (**d**–**f**) two-mode BHF case.

Figure 16 shows the numerical results of the optimal VBHF trajectory cases, resulting in a maximum stress of 388 MPa, maximum effective strain (PEEQ) of 0.41, minimum thickness of 0.86, and no occurrence of defects. Thickness distribution and PEEQ show correlation, and anisotropic distribution concentrates in PEEQ gradient corner regions and flange regions, resulting in more thinning caused by the higher PEEQ. The FLD results confirm that all interior portions are within the safe regions. The application of the two-segmented mode yielded similar improvements, emphasizing the efficacy of this approach in enhancing formability.

**Figure 17:**
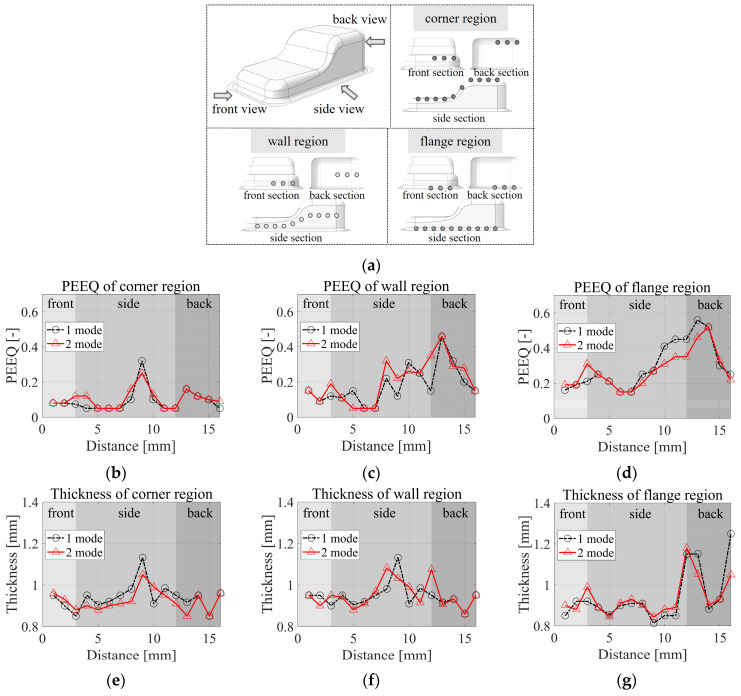
(**a**) Definition of measured points in defined sections and regions, (**b**–**d**) PEEQ, and (**e**–**g**) thickness distribution of region distance from front wall to back wall of oil pin product.

To measure the partial variability inherent in the molding outcomes, specific quantification was conducted at intervals of 50 mm along the oil pin product’s corners, walls, and flanges in the front, side, and back sections. This involves the designation of 3, 10, and 3 stationary points, respectively, for rigorous assessment. Figure 17a shows the definition of measured points in specified sections and regions, and therefore, Figure 17b,c show the PEEQ and thickness distribution of the region distance from the front wall to the back wall of the oil pin product via the optimal VBHF trajectories in a comprehensive and comparable way. The total variation of the coefficient of variation of thickness and PEEQ are 34.04% and 91.07%, respectively. The thickness increases in the middle of the flange region of the molded part around 10 mm on the side section, which corresponds to the corner region of the molded part. In addition, the thickness of the corner region of the molded part decreases as the flange region increases.

The strain distribution is anisotropic, meaning that the strain varies in different directions. The region around the center and bottom of the oil pan experiences higher plastic strain. This is because these areas undergo substantial stretching and deformation as the material is drawn into the die. The corners and edges of the oil pan may experience higher plastic strain compared to the central region. These areas often undergo localized stretching and deformation, leading to increased strain. Thus, in these statistics, the thickness increases in the middle of the flange area of the molded product, and the thickness of the flange area corresponding to the corner area of the stamped product increases. In addition, if the flange area increases, the thickness of the corner area of the stamped product decreases. Moreover, fluctuations form fluctuations implying inappropriate application of BHF.

Optimization results decrease the occurrence of some defects compared to the average results. Through comparison, it was found that the two-segmented mode case material did not accumulate at the center during the stamping process; thus, the corners might prevent the thinning as material flows toward the center, causing elongation and reduced thickness. Meanwhile, the side walls, with even material flow, may display a more relatively uniform thickness, which might cause decreased thickness in converging regions and thickening where material diverges. The side walls are subjected to a greater plastic strain compared to the center region, which results in significant stretching and deformation of the material as it is extruded into the die. Comparative results show that in the segmented mode, the material does not accumulate in the center during the stamping process, and therefore, the thickness is relatively uniform, whereas the thickness decreases in the converging region.

By adopting the coefficient of variation of the relative variability measure as the material flowing standard for assessment, the function for the coefficient of variation can be depicted as follows:

(12) $$CV=\frac{\varepsilon }{\eta }\times 100$$

where the mean deviation is denoted by ε, and the standard deviation is denoted by η. This method is widely used to evaluate changes in relative variability measurements by generalized statistical means [61,62,63,64]. In this study, a high coefficient of variation in the thickness or strain rate curve means that the material flow state is less mobile; that is, the probability of defects occurring is higher than the ideal state. For example, excessive lower material flow state regions could course failure defects such as tearing, while the excessive higher material flow state could cause local wrinkling.

**Figure 18:**
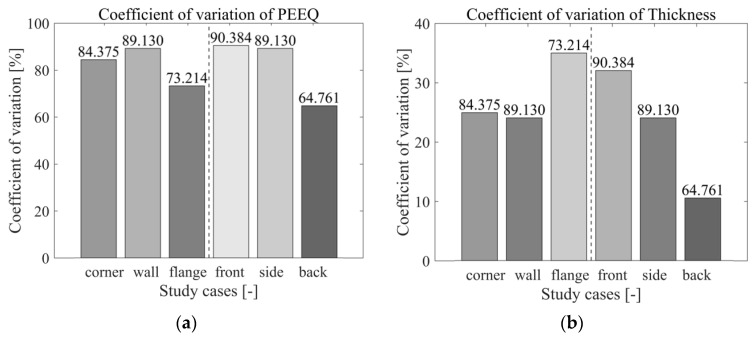
Coefficient of variation result of (**a**) PEEQ and (**b**) thickness of each determined study case.

The variations in individual coefficients within specified sections and regions, depicted in Figure 18, reveal consistent deviations in both the corner and wall areas. To enhance material flow, a lubrication body should be introduced in these flange areas. Notably, the back surface experiences greater fluctuations in thickness and strain rate compared to the front surface. Further structural design considerations, such as adjusting punch edge radius or mold gap, contribute to improved material flow.

## 7. Discussion

Metal components with precision shapes are fabricated using the stamping process, yet challenges persist in accommodating design changes. The developed DNN-GA-MCS integrated defect occurrence data collected from the stamping process to compare fracture characteristics for various Blank Holder Forces (BHF) and derived response surface models (RSMs). This method combined machine learning with a global search algorithm and presented an optimized inherent mechanism and interpretable transition.

Our research delves into the field of numerical studies concerning the forming processing, material properties, and the performance of materials. A deep neural network (DNN) constructing the response surface accurately approximates highly nonlinear coupling relationships between process variables, allowing for incremental learning. The Nondominated Sorting Genetic Algorithm-II (NSGA-II) optimization process employed a sequential sampling strategy, enabling global and local optimization simultaneously. Furthermore, Monte Carlo simulation (MCS) analysis of the Pareto-optimal sets improved forming processes from a stability perspective. Design constraints defined by material failure during forming processes were rigorously ensured and evaluated using the FLD evaluative framework. The discontinuous Pareto boundary is identified, obtaining optimal VBHF combinations where no failure, springback, or wrinkles are observed. Our study endeavors to make cutting-edge contributions in several key areas:

- Exploration of novel processing methodologies, exemplified by the stamping process with variable blank holder force (BHF) trajectories. Advancement of characterization, analysis, and modeling techniques to better understand and predict material behavior in stamping process parameter variables to uncover their unique properties and potential applications.
- The determination of optimal BHF trajectories was achieved through a surrogate model methodology that integrated deep neural network, genetic algorithm, and Monte Carlo simulation (DNN-GA-MCS) methodologies.
- The proposed approach utilized VBHF trajectories, which adjusted the BHF trajectories in the stamping cycle, thereby enhancing formability and mitigating the incidence of failure, springback, and wrinkling defects.
- The deep neural network (DNN) model was employed to address the intricate and nonlinear characteristics of the forming process. It is designed to approximate the functional relationship between defects and the trajectories of complex BHF, thereby constructing an approximated surface for analysis.
- The design constraint, defined as the failure of the sheet during the stamping process, was quantitatively evaluated employing the FLD based on the GISSMO damage model to ensure its rigorous assessment.
- In the proposed two-segmented mode VBHF case application, the average value of three defects improved by 12.62%, and the total quantity of VBHF was reduced by 14.07%. Meanwhile, compared with the average value of training sets, improvements of 18.89%, 13.59%, and 14.26% were achieved in failure, wrinkling, and springback, respectively.
- It was found that some further considerations of the structural design can be determined by using statistical methodologies. In the proposed two-segmented scenario, the back section of the oil pan sheet material exhibits more material flow fluctuation variations in thickness and strain rate in comparison to the front section.

These findings highlighted the practical implications of the methodology and demonstrated the efficacy of the surrogate model methodologies based on the DNN-GA-MCS approach in providing robust and reliable process parameter selections for intricate engineering applications.

However, certain limitations persist. Firstly, interpreting results from anisotropic FEA models can be challenging, while FEA is a valuable tool for analyzing anisotropic materials, it requires careful attention to the accurate definition and input of material characteristics. To understand and predict material behavior, advanced characterization used the Yld2004-18p yield function which accurately captures the anisotropic behavior of the alloy through 18 parameters. Some of the issues related to the accuracy of material coefficients, numerical stability and convergence, and mesh sensitivity were addressed, yet boundary condition sensitivity, temperature effects, and environmental effects with less impact were not involved. Secondly, DNN, GA, and MCS are all computationally intensive and require high resources. Combining them may result in high computational costs in terms of time and resources. When combined with the exploratory nature of GA, the risks of robustness and generalization become even more complex. Lastly, implementing a hybrid approach that combines DNN, GA, and MCS can be complex and require high specialized knowledge in all three areas. These are particularly challenging in the presence of high-dimensional data and complex fitness landscapes.

## 8. Conclusions

In the field of sheet metal forming, persistent challenges including failure, wrinkling, and springback present significant obstacles that require innovative solutions. This study introduced an innovative methodology specifically designed to address and mitigate these defects, which includes the utilization of a deep neural network (DNN) for advanced response surface model characterization, a genetic algorithm (GA) integrated with a complex model for multi-objective optimization, and Monte Carlo simulation (MCS) for validating the robustness of the results. Through meticulous analysis, several key findings and conclusions were derived:

- The VBHF trajectory displayed intricate behavior, notably with the emergence of discontinuous Pareto-optimal sets, and affirmed the advancement of the optimization method application.
- The response surface model effectively delineated the interrelations between the various parameters and their impacts on the outputs.
- VBHF trajectories were categorized based on specific goals: the minimization of defects, reduction in springback, and limitation of wrinkling, with their effectiveness confirmed through the Forming Limit Diagram (FLD).
- The FLD based on the GISSMO damage model offered an evaluation framework for a comprehensive understanding of sheet failure dynamics during forming.
- Numerical outcomes highlighted the substantial enhancements in the formed oil pan product quality: improvements of 18.89%, 13.59%, and 14.26% were observed in failure, wrinkling, and springback, respectively. The total quantity of VBHF was reduced by 14.07%.
- With increasing complexity in parameter design, an evolution in the derivation of optimal designs was anticipated. Nevertheless, the stability provided by MCS ensured the accuracy of precise outcomes. The generated datasets from the integration of the DNN, GA, and MCS served as valuable references for future industrial applications.

In conclusion, this study underscored the effectiveness of the integrated DNN-GA-MCS methodology in addressing the challenges associated with sheet metal forming, with the intricate process parameters represented by the VBHF trajectory proving to be particularly promising.

## Figures and Tables

**Table 1:** Representative literature of blank holder force (BHF) optimization.

Literature	Method	Input	Output
Srirat et al., 2012 [15]	LHD, RBF, SAO	BS, VBHF, traj	Earing
Kitayama et al., 2017 [17]	LHD, RBF, SAO	S-VBHF, BS, traj	Failure, wrinkling
Kitayama et al., 2017 [18]	LHD, RBF, SAO	VBHF, BS, traj	Failure, wrinkling
Kitayama et al., 2018 [19]	DOE, LHD, RBF, SAO	VBHF, BS, traj	Failure, wrinkling
Feng et al., 2018 [21]	LHD, MOABC, SPA	VBHF, traj	Failure, wrinkling, springback
Feng et al., 2019 [22]	LHD, SVR	VBHF, traj	Failure, wrinkling
Li et al., 2019 [16]	BPNN, MSE	CBHF	Thinning, thickness
Zhai et al., 2019 [23]	BBD, Kriging, MBC GA	vp, μ, DS, CBHF	Springback
Xie et al., 2019 [24]	LHS, SNRBF, NSGA-II, GRA	CBHF, traj	Thickening, thinning
Tran et al., 2021 [20]	DNN, GA	S-CBHF, DS	Earing, thickness
Yu et al., 2024 [27]	PMOO, SNTO	forming temperature, BHF	Thinning, springback
Jiang et al., 2024 [28]	LHD, Kriging, QO-Jaya	VBHF, traj	Failure, wrinkling
Guo et al., 2024 [10]	ANOVA, DOE, DNN, NSGA-II	S-VBHF, traj, DBFS, μ	Failure, wrinkling, springback
This study	LHD, DNN, NSGA-II	S-VBHF, traj	Failure, wrinkling, springback

Abbreviations: Latin hypercube design (LHD); radial basis function (RBF); sequential approximate optimization (SAO); blank shape (BS); trajectory (traj); segmented and variable blank holder force (S-VBHF); design of experiment (DOE); multi-objective artificial bee colony (MOABC); simultaneous perturbation approximation (SPA); support vector regression (SVR); back propagation neural network (BPNN); Box–Behnken design (BBD); model-based calibration (MBC); punch velocity (vp); sharing niching radial basis function (SNRBF); grey relational analysis (GRA); probabilistic multi-objective optimization (PMOO), sequential number-theoretic optimization (SNTO); quasi-oppositional Jaya (QO-Jaya).

**Table 2:** Representative literature of prediction of Forming Limit Diagrams using machine learning.

Literature	Method	Input	Output
Ali Derogar et al., 2011 [30]	ANN (3-4-2)	punch stroke, LDR, oil pressure	*ε*_2_, *ε*_1_
Paul, S. K. et al., 2016 [42]	regression equation	UTS, *n*, *r*, *t*, EU	*ε*_2_, *ε*_1_, FLC_0_
Chheda et al., 2019 [28]	SVR, GBR, NN (-)	CC, *T_h_*, *t_h_*, *T_i_*, *T_o_*, *t_r_*, *t_C_*, *T_C_*, *t_ag_*, *n*, *r*	*ε*_2_, *ε*_1_
F P Finamor et al., 2021 [41]	NN (-)	YS, UTS, EU, EL, R, *n*, *t*,	*ε*_2_, *ε*_1_
CG Dengiz et al., 2023 [29]	ANN (6-15-22-3)	YS, UTS, *ε*, *K*, *n*, *t*,	*ε*_2_^u^, *ε*_1_^u^, FLC_0_, *ε*_2_^b^, *ε*_1_^b^
SPSS Sivam et al., 2023 [40]	BR, LM, ANN	*t*, forming rates	*ε*_2_, *ε*_1_
This study	DNN (100-600-100-50)	Fracture locus	*ε*_2_, *ε*_1_

Abbreviation: material thickness (*t*), yield stress (YS), ultimate tensile strength (UTS), strength coefficient (*K*), strain hardening exponent (*n*), uniform elongation (*ε*), negative (*u*) and positive (*b*), uniform elongations (EU), total elongations (EL), homogenization time (*t_h_*), chemical composition (CC), homogenization temperature (*T_h_*), entry temperatures (*T_i_*), exit temperatures (*T_o_*), thickness reduction (*t_r_*), CASH time (*t_C_*), CASH temperature (*TC*), aging time (*t_ag_*), limit draw ratio (LDR).

**Table 3:** Anisotropy parameters of Yld2004-18p coefficients for AA6014-T4.

C′_12_	C′_13_	C′_21_	C′_23_	C′_31_	C′_32_	C′_44_	C′_55_	C′_66_
0.785	0.684	0.725	1.435	0.805	0.901	1.026	1.041	0.901
C″_12_	C″_13_	C″_21_	C″_23_	C″_31_	C″_32_	C″_44_	C″_55_	C″_66_
1.124	1.116	1.036	0.804	0.523	0.415	0.726	0.813	0.961

**Figure A1:**
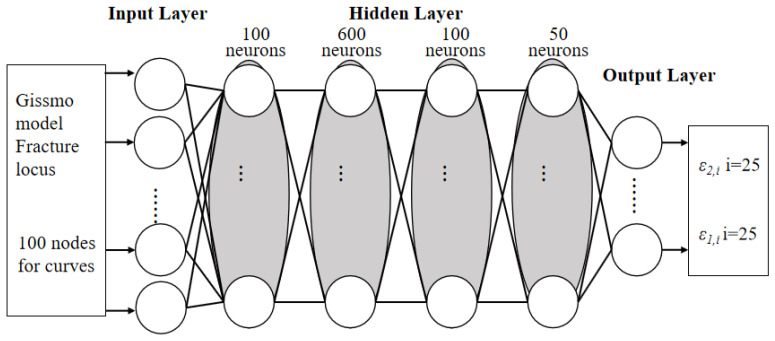
Schematic diagram of the Forming Limit Diagram DNN modeling.

**Figure A2:**
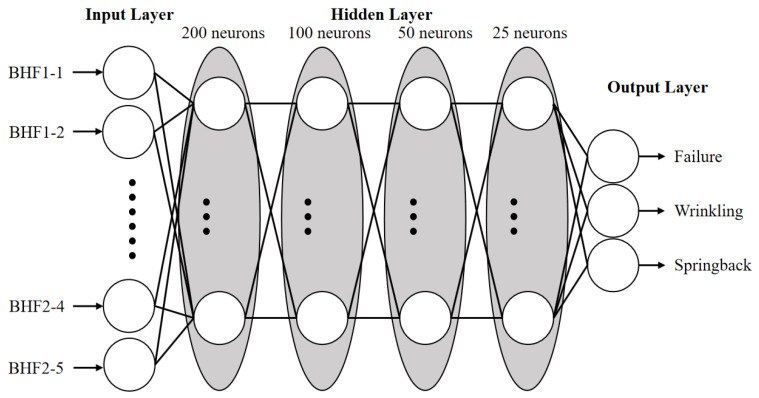
Construction schematic diagram of the variable blank holder force trajectory DNN modeling.

**Figure A3:**
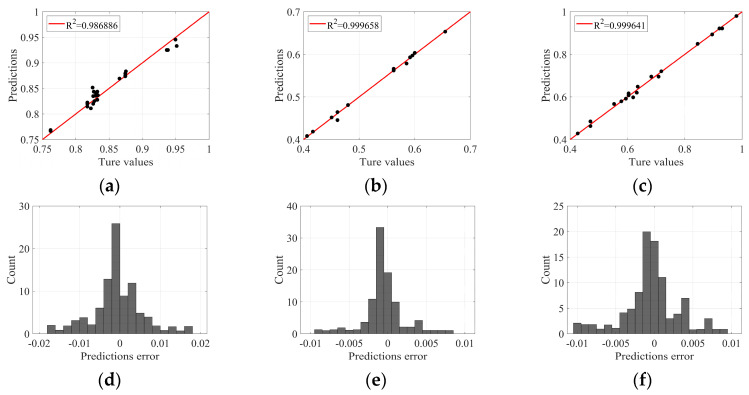
DNN validation results, showing connection between predicted and true values, approximation error, and R-squares. Results are segregated for training dataset ((**a**–**c**): springback, wrinkling, failure) and test dataset ((**d**–**f**): springback, wrinkling, failure).

**Figure A4:**
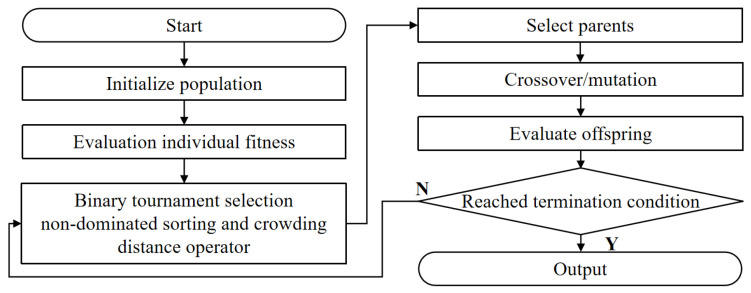
Schematic diagram of NSGA-II algorithm workflow.

**Figure A5:**
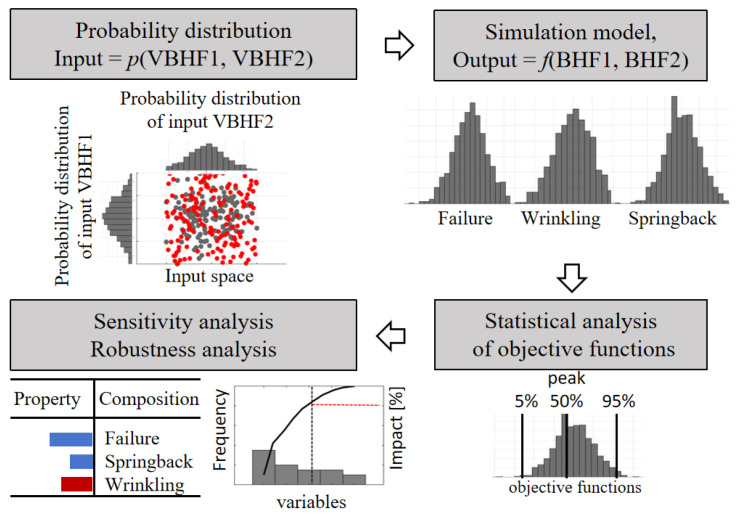
Workflow of Monte Carlo simulation.

## Data Availability

The data presented in this study are available on request from the corresponding author. The data are not publicly available due to privacy.

## Appendix A. Deep Neural Network (DNN) Modeling for Prediction of Forming Limit Diagram

To form the training data for the machine learning model, fracture locus curves were extracted by performing finite element analysis on six types of specimens as normal tensile specimens and the geometries shown in the figure above. The fracture locus curves were divided into 100 parts up to a certain displacement so that the input array could be the same. A DNN model to predict the major and minor strain from the fracture locus curves was constructed as shown in Figure A1. The input layer consists of 100 nodes. The hidden layer is composed of four layers, each of which is composed of 100, 600, 100, and 50 nodes. The output layer consists of 50 nodes and outputs the major and minor strain. Adam optimizer was used to train the DNN model, the learning rate was 0.0002, and the training was performed for a total of 500 epochs. After training the DNN model, the major and minor strains were derived using the fracture locus curves of the hybrid experimental-numerical procedure as an input.

RMSE (root-mean-squared error) to evaluate the accuracy of molding limit prediction is defined as the following equation, and as a result of the evaluation, it was confirmed that the machine learning model properly predicts the molding limit with 91.29%.

(A1) $$RMSE=\sqrt{\frac{1}{n}\sum_{i=1}^{n}{\left(y_{i}-\hat{y_{i}}\right)}^{2}}$$

where Ŷi represents the estimated value of Y, Yi signifies the value of Y, and n denotes the total number of samples.

## Appendix B. Variable Blank Holder Force Trajectory Deep Neural Network (DNN) Modeling

This research employed a DNN as a metamodel to approximate the RSM model, especially at critical points in the design space [65,66]. The DNN had an input layer with 10 neurons representing sub-stroke steps of VBHF, and an output layer with 3 neurons for failure, wrinkling, and springback. Referring to the previous research, model performance could be enhanced through the implementation of deeper and wider networks [67,68,69], and a DNN construct with five hidden layers was adopted. To improve model performance, hidden layers with varying numbers of neurons in each layer (128, 256, 512, 256, and 128), as showen in Figure A2. Rectified Linear Unit (ReLU) activation functions were used in the hidden layers to present nonlinearity and enhance feature representation [70,71]. This setup has been successful in nonlinear regression tasks, allowing for modeling complex relationships between input and output variables [72,73]. The construction of the DNN used in the study is shown in Figure 16. The implementation was performed using the Keras framework on TensorFlow.

To augment the ability to generalize to novel data instances, 90% of datasets were for training and 10% for testing to ensure the establishment of substantial and representative training datasets for the DNN model. The incorporation of the Adam optimizer, with a learning rate set at 0.001 [74,75], a batch size of 1000, and the execution of 1000 epochs, sped up the convergence process and enhanced the generalization of the network [76,77]. The precision in approximating values was achieved by the trained DNN model, as indicated in Figure A3. The predicted accuracy was obtained as a global MAE of 1.89 × 10^−5^ and MSE of 3.2 × 10^−3^, which could depict a significant alignment between the predicted and actual parameter values.

Two metrics, the mean absolute error (MAE) and the mean squared error (MSE), were used to evaluate the accuracies of the models. The MAE and MSE were employed to assess performance and residual variance by indicating the average magnitude of residuals and providing overall evaluations, respectively. The mathematical formulas used for calculating these indicators are as follows:

(A2) $$MSE=\frac{1}{n}\sum_{i=1}^{n}{\left(y_{i}-\hat{y_{i}}\right)}^{2}$$

(A3) $$MAE=\frac{1}{n}\sum_{i=1}^{n}\left|y_{i}-\hat{y_{i}}\right|$$

where Ŷi represents the estimated value of Y, Yi signifies the value of Y, and n denotes the total number of samples.

## Appendix C. Nondominated Sorting Genetic Algorithm-II (NSGA-II)

The NSGA-II algorithm was used to optimize the complexity involved in resolving the optimal VBHF process parameters. The NSGA-II algorithm was used to find non-inferior solutions within the Pareto optimal set of the multi-objective problem. This complexity stems from the conflicting trade-offs associated with having to minimize three objective defect functions. As shown in Figure A4, within the framework of a genetic algorithm, NSGA-II incorporated a survival selection process to the specific needs of the optimization problem. Solutions were categorized according to their non-dominant position, thus revealing trade-offs between objectives. The selection process then utilized the crowding distance, a method for evaluating solution diversity. Each individual in the population was defined by design variables, including the ten trajectories of the VBHF. The evaluation criteria involve three defects: failure, wrinkling, and springback. The optimization procedure was executed for more than 200 iterations. The NSGA-II implementation utilized the TensorFlow based on the Keras framework.

## Appendix D. Workflow of Monte Carlo Simulation (MCS)

Figure A5 illustrates the procedural steps involved in conducting a Monte Carlo simulation. The specific VBHF variables were first identified, and a Monte Carlo simulation model was created that accurately represented the input space system. Subsequently, random inputs were generated based on specified probability distributions for each variable. These generated inputs were then fed into the model to generate frequency results. The results were integrated to assess probabilities and facilitate decision-making by elucidating the effects of uncertainty on material behavioral properties.
